# Noninvasive and Real-Time Plasmon Waveguide Resonance Thermometry

**DOI:** 10.3390/s150408481

**Published:** 2015-04-13

**Authors:** Pengfei Zhang, Le Liu, Yonghong He, Yanfei Zhou, Yanhong Ji, Hui Ma

**Affiliations:** 1 Shenzhen Key Laboratory for Minimal Invasive Medical Technologies, Institute of optical imaging and sensing, Graduate School at Shenzhen, Tsinghua University, Shenzhen 518055, China; E-Mails: zhangpf14@mails.tsinghua.edu.cn (P.Z.); zhou-yf12@mails.tsinghua.edu.cn (Y.Z.); mahui@tsinghua.edu.cn (H.M.); 2 Department of Physics, Tsinghua University, Beijing 100084, China; 3 Institute of Green Chemistry and Energy, Graduate School at Shenzhen, Tsinghua University, Shenzhen 518055, China; E-Mail: liu.le@sz.tsinghua.edu.cn; 4 MOE Key Laboratory of Laser Life Science & Institute of Laser Life Science, South China Normal University, Guangzhou 510631, China; E-Mail: jiyh@scnu.edu.cn

**Keywords:** plasmon waveguide resonance, thermometry, thermo optic effect, optical temperature sensor

## Abstract

In this paper, the noninvasive and real-time plasmon waveguide resonance (PWR) thermometry is reported theoretically and demonstrated experimentally. Owing to the enhanced evanescent field and thermal shield effect of its dielectric layer, a PWR thermometer permits accurate temperature sensing and has a wide dynamic range. A temperature measurement sensitivity of 9.4 × 10^−3^ °C is achieved and the thermo optic coefficient nonlinearity is measured in the experiment. The measurement of water cooling processes distributed in one dimension reveals that a PWR thermometer allows real-time temperature sensing and has potential to be applied for thermal gradient analysis. Apart from this, the PWR thermometer has the advantages of low cost and simple structure, since our transduction scheme can be constructed with conventional optical components and commercial coating techniques.

## 1. Introduction

Temperature sensing is of importance in several fields [1], such as the microfluidic research [2], molecular interaction analysis [3] and clinical medicine [4]. Owing to the attractive advantages of being pollution free, having electric immunity and being of a compact size, the optical temperature sensors were studied and constructed by employing fluorescence dye [5], interferometer [6], microfiber knot resonator [7], optical microspheres [8], whispering-gallery mode dielectric resonator [9], Goos-Hänchen effect [10] and surface plasmon resonance (SPR) techniques [11]. The SPR thermometers permit noninvasive and real-time temperature sensing and have a simple sensor structure [11,12,13]. They have broad application prospect with the aim to provide an economical and convenient thermometer module based on the fact that the SPR sensors have been widely used for label-free refractive index (RI) related measurements [3,14,15,16,17]. Using the evanescent field of the surface plasmons, which propagate along the interface of two materials with real dielectric constants of opposite signs, the SPR thermometers probe the thermo optics (TO) coefficients of the materials for temperature sensing [11]. In the past decade, the temperature effect on the SPR sensors was studied at the bare metal film [12,18], metal-semiconductor interface [19], gold-water interface [20] and nanoparticles-dielectric interface [21]. SPR thermometers were constructed for transient thermal field analysis [22], microfluidic thermometry [13], *in situ* thermometry [23] and thermal gradient analysis [24]. However, because of the background TO effect of metal film, the sensitivity of the SPR thermometers is usually limited to the level of 0.1 °C, which is achieved by using comprehensive analyses to measure the intrinsic temperature sensitivity [23]. The dynamic range is usually limited to about 10 °C achieved by using the angle interrogation [24] that has the widest dynamic range among four types of interrogations of the SPR sensors [25].

One effective method to improve the performance of SPR sensors is coating a dielectric layer on the metal film to enhance the evanescent field [26,27]. When the dielectric layer has both appropriate RI and thickness, the surface plasmons are coupled to the waveguide mode of the dielectric layer to construct the plasmon waveguide resonance (PWR) sensor, which is another kind of surface plasmon based optical sensors [28]. It has been pointed out that the performance of the PWR sensors can exceed that of the SPR sensors owing to enhancement of evanescent field and sharp reflectance spectrum in RI sensing applications [17,27,29]. Due to the small thermal conductivity of dielectric layers, the heat caused by wave propagation at the metal dielectric interface has less influence on the fluid RI in the PWR sensors [29]. Considering these advantages and the fact that the dielectric layer can protect the metal film against mechanical or chemical deterioration [30], we assume that the dielectric layer may have the ability to play the role of thermal shield of the metal film, which can improve the sensitivity and dynamic range of SPR based thermometers.

In this article, we report the PWR thermometer for noninvasive and real-time temperature sensing. The characteristics of the SPR and PWR sensors are compared, and the thermometry principles of the SPR and PWR sensors are analyzed. In a next step, the TO coefficient and cooling process of water are measured to test the system characteristics in the temperature measurement. In these experiments, we demonstrate that the PWR thermometer does not only have the advantages of the SPR thermometers, but also permits accurate temperature sensing in a wide temperature range because it can be free of background thermo optic effect of the sensing structure and has an enhanced evanescent field.

## 2. Experimental Section

### 2.1. Design Consideration

The well-known Kretschmann’s analysis [31] is employed in this study. A gold (Au) film, which is commonly used in the SPR sensors [3], is employed as the surface plasmon active layer. A magnesium fluoride (MgF_2_) film, which is easy to prepare and does not need an adhesion layer with a Au film [27], is employed as the dielectric layer. Considering that the transverse magnetic (TM) polarization has a large sensitivity to the RI variations [32] and better performance than the transverse electric (TE) polarization in the RI sensing applications [27,29], the performance of the PWR thermometer in the TM mode is the focus of this study. Using the multiple reflectance theory and Fresnel formula, the total reflectance in the TM mode can be calculated with:

(1) $$R={\left|\frac{r_{12}+r_{2\ldots k}\exp(2i\beta_{2})}{1+r_{12}r_{2\ldots k}\exp(2i\beta_{2})}\right|}^{2},r_{2\ldots k}=\frac{r_{23}+r_{3\ldots k}\exp\left(2i\beta_{3}\right)}{1+r_{23}r_{3\ldots k}\exp\left(2i\beta_{3}\right)}$$

(2) $$r_{i,j}=\frac{X_{i}-X_{j}}{X_{i}+X_{j}},X_{i}=\frac{\varepsilon_{i}}{k_{iz}}$$

where *k* is the total layer number, ε*_j_* and *d_j_* are the dielectric constant and thickness of *j*th layer, respectively, ω and θ are the angular frequency and incident angle of the incident light, respectively, and *c* is light speed in the vacuum [33,34].

**Figure 1:**
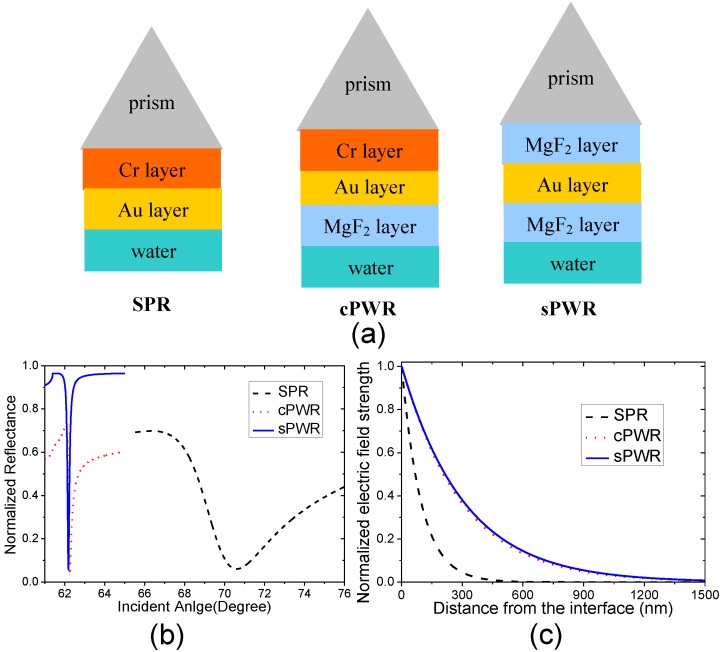
(**a**) Schematics of the surface plasmon resonance (SPR), conventional plasmon waveguide resonance (PWR) (cPWR), symmetrical PWR (sPWR) structures; (**b**) Angular reflectance spectra measuring water; (**c**) Normalized electric field strengths of the evanescent fields inside the water are plotted against the distance from the film/water interface. The refractive indices of the prism, Cr, Au, MgF_2_ and water we used are 1.515, 3.44 + 4.34i, 0.13 + 3.65i, 1.38 and 1.33. The thicknesses of Cr, Au and MgF_2_ layers on contact with the water are 3 nm, 40 nm, and 800 nm. The MgF_2_ layer sandwiched between the prism and gold film in the sPWR structure is 500 nm. The incident wavelength is 632.8 nm.

In this study, we employ the symmetrical PWR (sPWR) structure, which is also called the symmetrical optical waveguide structure [35] that has been used for the RI sensing applications [17]. As shown in Figure 1a, another MgF_2_ film is sandwiched between the prism and gold film in the sPWR structure instead of the adhesion layer, which is usually the chromium (Cr) film (in this study) or titanium film used in the conventional PWR (cPWR) structure for available gold film firmness [27,29]. The angular reflectance spectra of the SPR, cPWR and sPWR sensors measuring the water are calculated and shown in Figure 1b. Combined with the method described by Hansen [36], the electric field strength distributions of the evanescent fields inside the water of the three structures are calculated and shown in Figure 1c. It can be seen from Figure 1 that the sPWR and cPWR sensors have similar evanescent field penetration depths and reflectance spectra, which are both longer and sharper than those of the SPR sensor. However, the angular reflectance spectrum of sPWR sensor has a higher extinction ratio than that of cPWR sensor, which can be duet to two reasons. One reason is that the long-range surface plasmon, which has a lower energy loss in the Au layer [37], is excited and coupled owing to the symmetrical condition [35]. The other reason is that the dielectric (MgF_2_) layer has a smaller light absorption coefficient compared with the metal adhesion layer. Considering these facts and the good performance in the RI sensing applications [17], the sPWR sensing structure is employed in this study to construct the PWR thermometer.

It is known that the waveguide sensors can also be constructed without the surface plasmon active layer, namely the metal film, which is configured in the photonic mode based on the attenuated total reflection. In this experiment, this type of sensor can be constructed using some materials with high RI, such as Si_3_N_4_ and TiO_2_. But the SPR and PWR sensors have distinct advantages in the measurement due to the sharper resonance dip and the fact that the spectral analysis can be easily used in the data processing of their signals, so the waveguide sensors using plasmonic mode, namely the PWR sensors are employed in this study.

The principles of SPR and PWR thermometers are similar regarding the measurement of the temperature dependent RI variation of samples within the evanescent field [11]. The relation between RI *n* and relative permittivity ε*_r_* for the dielectric can be shown as [38]:

(3) $$n=\sqrt{\varepsilon_{r}}$$

The relation between electronic polarizability α and relative permittivity ε*_r_* is:

(4) $$\alpha =\varepsilon_{0}\left(\varepsilon_{r}-1\right)$$

where ε*_0_* is the vacuum permittivity [39]. The electronic polarizability of dielectric molecules usually varies with the temperature [40], which leads to a temperature dependent RI variation, namely the so-called TO effect. Hence the temperature of materials can be measured by using a RI sensor. The relation between RI *n* and the temperature *T* of the water, which is used as the sample in this study, is shown as:

(5) $$\begin{array}{c} \frac{n^{2}-1}{n^{2}+2}\frac{1}{\rho^{*}}=a_{0}+a_{1}\rho^{*}+a_{2}T^{*}+a_{3}\lambda^{*2}T^{*}+a_{4}/\lambda^{*2}\\ +\frac{a_{5}}{\lambda^{*2}-\lambda_{UV}^{*2}}+\frac{a_{6}}{\lambda^{*2}-\lambda_{IR}^{*2}}+a_{7}\rho^{*2} \end{array}$$

(6) $$\rho^{*}=\frac{\rho }{\rho_{0}},\lambda^{*}=\frac{\lambda }{\lambda_{0}},T^{*}=\frac{T}{T_{0}}$$

where ρ is the density, λ is the electromagnetic wavelength, *T* is the absolute temperature, and ρ*_0_*, λ*_0_*, λ*_UV_*, λ*_IR_*, *T_0_*, *a_0_*, *a_1_*, *a_2_*, *a_3_*, *a_4_*, *a_5_*, *a_6_*, *a_7_* are constants [41]. Using Equations (5) and (6), the TO coefficient of water can be calculated to be in the order of 10^−4^ RIU/°C.

**Figure 2:**
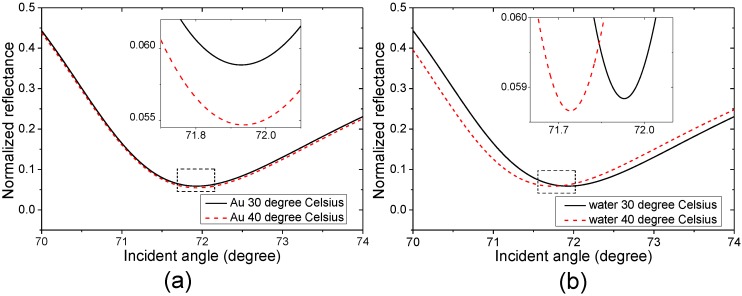
(**a**) Calculated SPR reflectance spectra when the temperature of the Au layer varies from 30 °C (the black solid line) to 40 °C (the red dash line) while the water temperature is maintained at 30 °C; (**b**) Calculated SPR reflectance spectra when the water temperature varies from 30 °C (the black solid line) to 40 °C (the red dash line) while the temperature of the Au layer is maintained at 30 °C. (Inset) the enlargement of the marked zones. At the temperature of 30 °C, the refractive indices of the Au layer and water are 0.1324 + 3.6544i and 1.3403 RIU, respectively. At the temperature of 40 °C, the refractive indices of the Au layer and water are 0.1358 + 3.6530i and 1.3389 RIU, respectively. The refractive indices of the prism and Cr are 1.515 and 3.44 + 4.34i. The thicknesses of the Cr and Au layers are 3 nm and 40 nm, respectively. The incident wavelength is 632.8 nm.

In the SPR thermometer, the TO effect of an optical glass prism with the TO coefficients in the order of 10^−6^ RIU/°C can be neglected [11,24]. But the TO effect of the Au film, which is in contact with the samples and a good thermal conductor, cannot be neglected. The dielectric constant ε of the Au film can be calculated with the Drude model:

(7) $$\varepsilon ={\left(n_{r}+in_{i}\right)}^{2}=1-\frac{\omega_{p}^{2}}{\omega \left(\omega +i\omega_{c}\right)}$$

where *n_r_* and *n_i_* are real and imaginary parts of RI, ω is the angular frequency of the incident light, and ω*_p_* and ω*_c_* are plasmon frequency and collision frequency of the electrons, respectively. The plasmon frequency can be calculated using:

(8) $$\omega_{p}\left(T\right)=\omega_{p}\left(T_{0}\right){\left[1+3\gamma \left(T-T_{0}\right)\right]}^{-\frac{1}{2}}$$

where ω*_p_(T_0_)* is the plasmon frequency at a reference temperature *T_0_*, and γ is the thermal linear expansion coefficient [42]. In the metal film, the electron-phonon and electron-electron scattering processes contribute to the temperature dependent RI variation and can be estimated with:

(9) $$\omega_{c}=\omega_{0}\left[\frac{2}{5}+\frac{4T^{5}}{\theta_{D}^{5}}\int_{0}^{\theta_{D}/T}\frac{z^{4}}{e^{z}-1}dz\right]+\frac{\pi^{4}\Gamma \Delta }{6hE_{F}}\left[{\left(k_{B}T\right)}^{2}+{\left(h\omega /4\pi^{2}\right)}^{2}\right]$$

where Γ is the Fermi surface average of scattering probability, Δ is the fractional Umklapp scattering, θ is the Debye temperature, *E_F_* is the Fermi energy, *h* is the Planck constant and *k_B_* is the Boltzmann constant [43,44]. Based on these equations, the TO coefficient of the Au film can be calculated as *dn_r_/dT* ≈ 3.4 × 10^−4^ RIU/°C and *dn_i_/dT* ≈ −1.4 × 10^−4^ RIU/°C. They are in the same order with the TO coefficients of water. In a next step, the negative effect of the background TO effect of the Au film on the measurement accuracy of SPR thermometers is estimated. The propagation constant of the evanescent wave *k_e_* can be given by the standard phase matching condition

(10) $$k_{e}=k_{inc}n_{p}\sin\theta$$

where *k_inc_* is propagation constant of the incident light wave, *n_p_* is the RI of the prism and θ is the incident angle. The evanescent wavelength in the SPR configuration shown in the Figure 1 can be estimated to be approximately 440 nm. At this wavelength, the water TO coefficient can be estimated to be approximately 1.4 × 10^−4^ RIU/°C when the temperature varies from 30 °C to 40 °C using Equations (5) and (6). Based on these considerations, the SPR angular reflectance spectra are calculated and shown in Figure 2a where the Au layer temperature varies from 30 °C to 40 °C and Figure 2b where the water temperature varies from 30 °C to 40 °C.

It can be seen from Figure 2 that the resonance angle, which is the incident angle affording the minimum reflectance intensity, shifts from 71.9295° to 71.9359° and the variation is 0.0064° when only varying the Au layer temperature, and from 71.9295° to 71.7410° and the variation is 0.1885° when only varying the water temperature. The temperature rise of the Au layer leads to the resonance angle shifting to a higher value, and the water temperature rise leads to the resonance angle shifting to a lower value. It can be seen that the resonance angle variation caused by the background TO effect of the Au layer is almost one-thirtieth of the variation caused by the water temperature variation. This has a deteriorating effect on the temperature measurement accuracy of the SPR thermometers, especially when used for measuring the temperature varying in a wide range.

**Figure 3:**
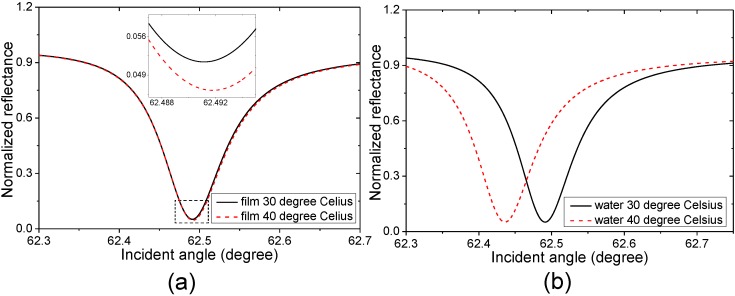
(**a**) Calculated sPWR reflectance spectra when the film temperature of the sensing structure varies from 30 °C (the black solid line) to 40 °C (the red dash line) while the water temperature is maintained at 30 °C; (**b**) Calculated sPWR reflectance spectra when the water temperature varies from 30 °C (the black solid line) to 40 °C (the red dash line) while the film temperature of sensing structure is maintained at 30 °C. (Inset) the enlargement of the marked zones. At the temperature of 30 °C, the refractive indices of the Au layer, MgF_2_ layer and water are 0.1324 + 3.6544i, 1.3804 and 1.3381 RIU, respectively. At the temperature of 40 °C, the refractive indices of Au layer, MgF_2_ layer and water are 0.1358 + 3.6530i, 1.38041 and 1.3367 RIU, respectively. The RI of the prism is 1.515. The thicknesses of Au layer and MgF_2_ layer in contact with the water are 40 nm and 800 nm, respectively. The MgF_2_ layer sandwiched between the prism and gold layer is 500 nm. The incident wavelength is 632.8 nm.

In the PWR thermometer, the MgF_2_ layer is in contact with the water. The thermal conductivity coefficient, specific heat capacity and density of the MgF_2_ are 0.035 cal/(s·cm·°C) [45], 1020 J/(kg·°C) and 3180 kg/m^3^ [46], respectively, and the MgF_2_ has a TO coefficient of approximately 1 × 10^−6^ RIU/°C [47], which is in the same order with that of optical glass and much lower than that of the Au film. Although the MgF_2_ layer is not a good thermal conductor and has a large specific heat capacity, which may cause the film temperature fluctuations of the sPWR sensing structure smaller than those of the SPR sensing structure, we analyze the worst case where the sPWR sensing structure has the same film temperature variation as the water. Using Equation (10), the evanescent wavelength in the sPWR configuration shown in the Figure 1 can be calculated to be approximately 473 nm. At this wavelength, the water TO coefficient can be estimated to be approximately 1.4 × 10^−4^ RIU/°C when the temperature varies from 30 °C to 40 °C using Equations (5) and (6). Based on these considerations, the angular reflectance spectra are calculated and shown in Figure 3a where the film temperature of the sPWR sensing structure varies from 30 °C to 40 °C while the water temperature is maintained at 30 °C, and Figure 3b where the water temperature varies from 30 °C to 40 °C while the film temperature of the sensing structure is maintained at 30 °C.

It can be seen from Figure 3 that the resonance angle, which is the incident angle affording the minimum reflectance intensity, shifts from 62.4911° to 62.4917° and the variation is 0.0006° when only varying the film temperatures of sPWR sensing structure, and it shifts from 62.4911° to 62.4359° and the variation is 0.0552° when only varying the water temperature. The temperature rise of the sensing structure leads to the resonance angle shifting to a higher value, and the water temperature rise leads to the resonance angle shifting to a lower value. It can be seen that the resonance angle variation caused by the background TO effect of the sensing structure is almost one percent of the variation caused by the water temperature variation. The negative effect of the background TO effect of sPWR sensing films is about five times smaller than that of the SPR sensing film. This indicates that the sPWR thermometer could suppress the negative effect of background TO effect significantly. The thermal expansion effect is usually the other interfering factor in the optical temperature sensing. However considering the fact that the linear thermal expansion coefficients of Au and MgF_2_ are both in the order of 10^−5^/°C [11,48], the film thickness variation caused by the thermal expansion effect can be neglected in both SPR and PWR thermometers. In summary, it is possible to construct a thermometer allowing accurate temperature sensing in a wide dynamic range using the PWR technique.

### 2.2. Sensor Chips

A polished BK7 glass substrate is firstly cleaned with a solution consisting of ethanol and diethyl ether in 1:1 ratio, then rinsed with deionized water (Milli-Q water) and dried with nitrogen. Then the substrate is sequentially coated with a 500 nm MgF_2_ film, a 40 nm Au film and an 800 nm MgF_2_ film to construct the sPWR sensor chip. The gold film is coated using magnetron sputtering with the film thickness measured by a quartz crystal oscillator thickness monitor. The substrate heating and bias voltage techniques are employed in the coating process to improve the Au film thickness uniformity and firmness [24]. The MgF_2_ film is deposited using evaporation coating with the film thickness measured by a step profiler. The MgF_2_ crystals are used as the evaporation materials and the weight is controlled for by the specific film thickness in the coating process [27]. In a next step, the sensor chip is placed on a BK7 prism with the RI matching oil (Cargille) to construct a sPWR sensor module.

### 2.3. Experimental Setups

**Figure 4:**
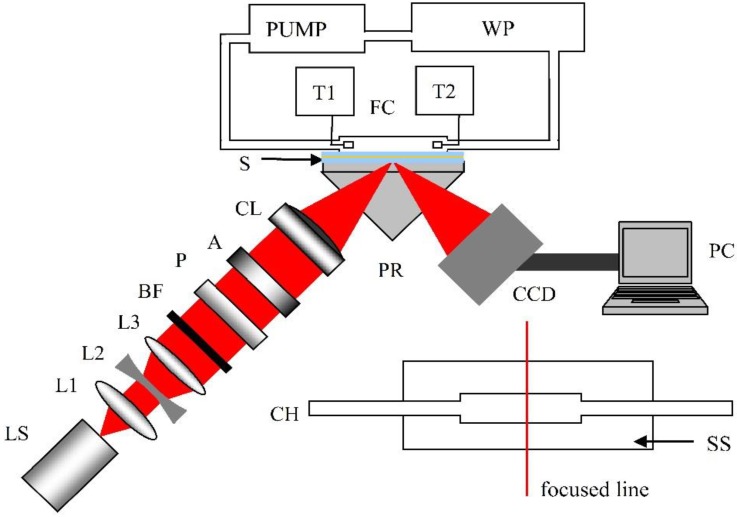
Schematic of the experimental system: LS, integrated light source; L1, convex lens; L2, concave lens; L3, convex lens; BF, bandpass filter; P, linear glass polarizer; A, rectangular aperture; CL, cylindrical lens; PR, prism; S, sensor chip; FC, flow cell; CCD, charge coupled device; PC, personal computer. WP, programmable water path; PUMP, peristaltic pump; T1, T2, digital thermocouple probes; CH, fluidic channel; SS, sensing surface.

A conventional one-dimensional imaging system is employed in this study as shown in Figure 4. The integrated light source (LS) employs a 25× objective lens to focus the light from a red light emitting diode with an electric power of 3W on a pinhole. Then the light is collimated by the collimation lenses consisting of two convex lenses (L1, L3) and one concave lens (L2) before being filtered by a bandpass filter (BF, center wavelength 632.8 nm, bandwidth 10 nm, Thorlabs), and p-polarized by a linear glass polarizer (P). An aperture (A) is used to acquire a rectangular light spot. After passing through a cylindrical lens (focus length 40 mm) with a vertical axis of symmetry, the light is focused to a narrow line on the sensing surface (SS) to construct an imaging channel. The sPWR module is configured in the Kretschmann manner with the attenuated total reflection method in this system. A polymethyl methacrylate flow cell (FC, 10 × 3 × 3 mm, 90 μL) is attached to the sensor chip for sample delivery to the sensing surface through the fluidic channel (CH). The reflection light beams are recorded by a charge coupled device (CCD, 2580 × 1912 pixels, 3.4 μm pixel size, ICX282AQ, Sony) for further data analysis in a personal computer (PC). The temperature control system is configured in the manner described in [24,49]. The sample continuously circulates through a programmable water bath (WP). A peristaltic pump (PUMP) circulates the water through the cell at a rate of about 10 mL/min. The temperature in the cell is measured by two digital thermocouple probes (T1, T2) with 0.1 °C resolution.

In the experiment, the resonance angle is represented by the corresponding pixel position affording the minimum intensity in the captured image. The facula after passing the rectangular aperture forms a square with the size of 10 × 10 mm^2^. Using the focus length of the cylindrical lens and considering the refraction effect of the prism, the angular range of the incident wedge light beam is approximately 8.13°. The reflectance light beam forms a rectangle with the size of approximately 52 × 10 mm^2^ when captured by the CCD. Considering the pixel size, the angular resolution of the imaging system can be calculated to be 5.3 × 10^−4^ degree/pixel. In this imaging system, the left area in the CCD sensing area, where the pixel number is represented by small values in the captured images, records the reflectance light beam with a large incident angle. This is due to the CCD characteristics.

## 3. Results and Discussion

### 3.1. RI Test

The deionized water and the glucose solutions with different indices are used to test the sensor response to the RI change. The relationship between RI *n* and concentration *C* of the glucose solution is *n* = *n* (water) + 1.515 × 10^−4^ × *C*, where *C* is the concentration in grams per liter. The experimental temperature is approximately 27 °C and the temperatures of the solutions are controlled to be 27 °C. The evanescent wavelength in the sPWR sensor can be estimated to be approximately 473 nm with Equation (10), so the water RI can be calculated to be 1.3384 using Equations (5) and (6). In this experiment, the resonance angle is represented by the corresponding pixel position affording the minimum intensity. To suppress the deviation caused by the response non-uniformity of the CCD, a hundred adjacent rows in one image are averaged to acquire the angular spectrum measuring the samples flowing through corresponding area. A ten order polynomial curve fitting method is applied to reduce the noise for determination of the pixel position of the resonance angle [50]. The water and the glucose solutions with concentrations of 1, 2, 3, 4, 5, 6 and 7 g/L are measured serially. Fifty images are captured with an exposure time of 600 ms for each sample and the capture time interval is 5 s.

**Figure 5:**
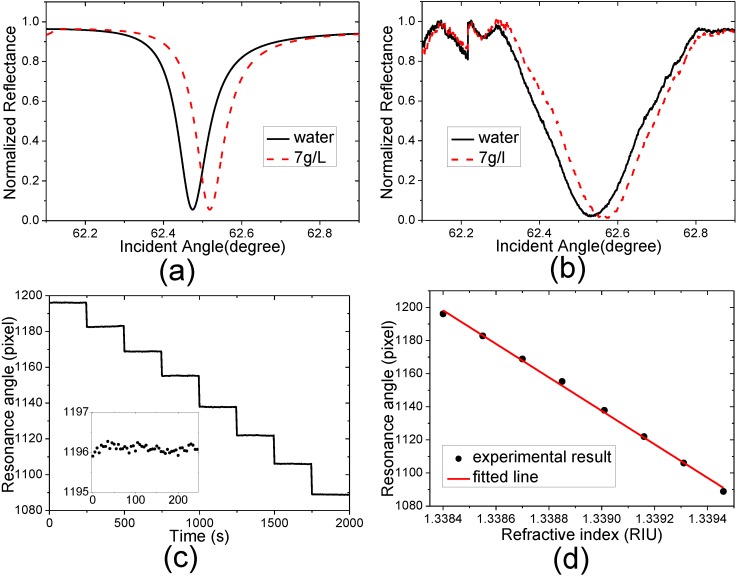
(**a**,**b**) Calculated, experimental angular reflectance spectra measuring the water and glucose solution with a concentration of 7 g/L; (**c**) Measured resonance angle as function of time, inset: enlargement of the function measuring the water at the time interval from 0 s to 250 s; (**d**) Averaged measured resonance angle as function of the refractive index. The error bars representing the standard errors lie within the data characters.

Figure 5a,b shows the theoretical and experimental angular reflectance spectra measuring the water and glucose solution with a concentration of 7 g/L. The measured resonant angles as the function of time and averaged measured resonance angle as the function of RI are shown in Figure 5c,d, respectively. In this experimental setup, the pixel positions with larger values represent the smaller incident angle due to the system characteristics. The combined sensitivity factor (*CSF*) [51] and RI resolution σ [52] are employed to estimate sensor characteristics. These functions are shown as:

(11) $$CSF=SF\times \frac{R_{\max}-R_{\min}}{FWHM},\sigma =\frac{\sigma_{SO}}{SF},SF=\frac{\partial \theta }{\partial n}$$

where θ is the incident angle, *n* is the RI of sample, *R_max_* and *R_min_* are the maximum and minimum normalized reflectance, *FWHM* is the full width at half maximum, σ*_SO_* is the standard deviation of sensor output, and *SF* is the sensitivity factor. Based on these functions and Figure 5a,b, the theoretical and experimental *CSF*s of sPWR are approximately 381 and 206, respectively. It can be seen from Figure 5c,d that the sensor output response is linear for the RI and the sensitivity factor is 1.01 × 10^5^ pixels/RIU, namely 53.5 degree/RIU. The standard deviation of the baseline created by the deionized water, whose RI is free of evaporation effects, is 0.082 pixel, so the RI resolution of this sPWR sensor can be calculated to be 7.9 × 10^−7^ RIU. The performance of this sPWR sensor is compared with those of the cPWR sensor using an Au-MgF_2_ structure [27], the cPWR sensor using an Au-Silica structure and the SPR sensor [29] at the incident wavelength of 632.8 nm in the TM mode as shown in Table 1. In these studies, similar experimental methods are employed without optimization for either data process algorithm or optical system, which employs the high quality area detector [35] or new technique such as the polarization interferometry [25].

**Table 1:** Comparison of the PWR and SPR sensors’ characteristics at the incident wavelength of 632.8 nm in the transverse magnetic (TM) mode.

Sensor	Theoretical CSF (RIU^−1^)	Experimental CSF (RIU^−1^)	RI Resolution (RIU)
SPR	14	12	3.2 × 10^−6^
Silica-cPWR	37	38	2.3 × 10^−6^
MgF_2_-cPWR	167	142	9.3 × 10^−7^
sPWR	381	206	7.9 × 10^−7^

It can be seen that the RI resolution and theoretical and experimental *CSF*s of sPWR sensor are all better than those of SPR and cPWR sensors at the incident wavelength of 632.8 nm. This enhancement can be due to the extinction ratio improvement of the angular reflectance spectrum and enhanced evanescent field, which can be supported by the theoretical predictions shown in Figure 1. It should be noticed that the difference between the theoretical and experimental *CSF*s of the sPWR sensor is larger than that between the cPWR and SPR sensors, which can be explained by in two ways. One reason is more the complicated sensing film structure of the sPWR sensor, so the matching relation is more difficult to be preserved in the practical applications due to film quality fluctuation in the coating process. The other reason is that both the MgF_2_ layers have large film thicknesses, so the negative effect of the film thickness errors in the coating process will be more significant. Considering these, the sPWR sensor still shows its advantage in the RI sensing application, whose RI resolution of 7.9 × 10^−7^ RIU is at the best level that can be provided by the SPR sensor under the incident wavelength of 632.8 nm predicted by Piliarik and Homola [52].

### 3.2. Temperature Sensing

The temperature variations of water, a material widely used for research and applications, are measured to test the system characteristics in the temperature sensing application. The water RI has little deviation from the linear correspondence to the temperature in a wide temperature range [41], but its response can be considered to be linear for the temperature in a narrow temperature range [13,24]. In this experiment, we firstly control the water temperature varying from 30 °C to 40 °C every 2 °C to test the measurement accuracy of this system, and then from 30 °C to 80 °C every 10 °C to demonstrate the TO coefficient nonlinearity of water. The room temperature is set to be 30 °C using the air conditioner. The sensor outputs measuring the water at the temperature of 30 °C are used as the baseline. Fifty images are captured with the exposure time of 600 ms for each temperature and the capture time interval is 5 s.

**Figure 6:**
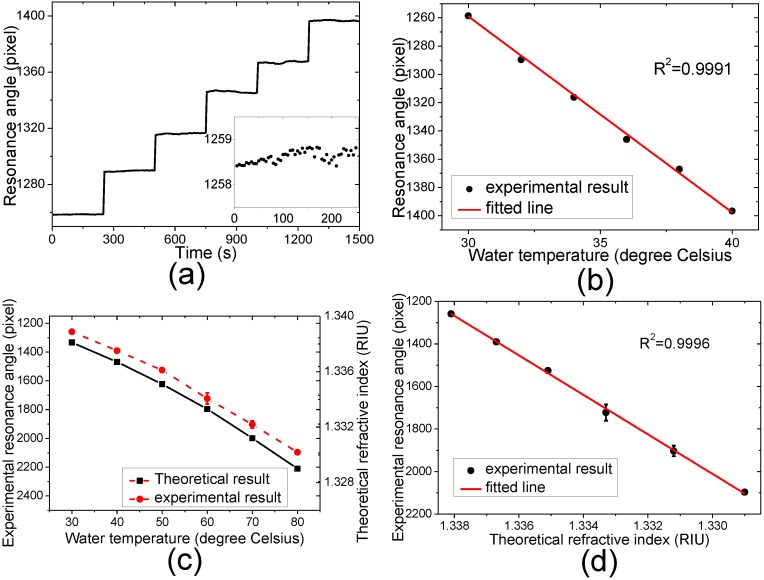
(**a**) Measured resonance angle as function of time, inset: enlargement of the function measuring the water at the temperature of 30 °C at the time interval from 0 s to 250 s; (**b**) Averaged measured resonance angle as function of the water temperature. The error bars representing the standard errors lie within the data characters; (**c**) Comparison between experimental resonance angles and theoretical refractive indices. The error bars represent the standard errors; (**d**) Measured resonance angle as function of the water refractive index at different temperature. The error bars represent the standard errors.

Figure 6a shows the measured resonant angles probing the water temperature varying from 30 °C to 40 °C every 2 °C as the function of time, and Figure 6b shows the averaged measured resonance angle as the function of the water temperature. The experimental results and theoretical refractive indices of water in the temperature range from 30 °C to 80 °C every 10 °C are shown in Figure 6c and the experimental results as the function of the theoretical refractive indices are shown in Figure 6d.

It can be seen from Figure 6a that the pixel position of the resonance angle shifts to the large value with temperature rising, which means the resonance angle shifts to a small value in this system when the water RI decreases. This is in agreement with the experimental results shown in Figure 5. It can be seen from Figure 6b that the sensor output responds linearly to the temperature, which means the water TO coefficient could be seen as a constant in this temperature range to estimate the thermometer characteristics in the temperature sensing applications. The measured TO coefficient in the temperature range from 30 °C to 40 °C is 13.86 ± 0.19 pixels/°C. After converted the pixel shift to RI using the sensitivity factor obtained in the RI test, the TO coefficient of water in this temperature range can be calculated to be (1.37 ± 0.019) × 10^−4^ RIU/°C. This is close to the calculated values of 1.40 × 10^−4^ RIU/°C for water using Equations (5) and (6) under the evanescent wavelength of 473 nm in this temperature range. The deviation may be because of the measurement errors and difference between the water quality in the experiment and calculations. In order to compare the temperature measurement sensitivity among various approaches, the temperature measurement sensitivity σ*_T_* is calculated according to

(12) $$\sigma_{T}=\frac{dT}{dp}\sigma_{p}$$

where *p* is the parameter being monitored during the measurement of the temperature variation, *T* is the temperature, and σ*_p_* is the available parameter resolution of experimental system [12]. In this experiment, the pixel position shift is the monitored parameter and the parameter resolution is the standard error of the baseline [52]. Considering the standard error of 0.13 pixels for the baseline shown in the insert in Figure 6a, the temperature sensitivity of this system can be calculated to be 9.4 × 10^−3^ °C, which is comparable to the recently reported high-resolution optical temperature sensor [10]. It can be seen that the standard error of sensor output in the temperature sensing is larger than that in the RI test, which should be caused by the noise of the temperature control system in the process of varying water temperature.

When the temperature range is wide, the water nonlinearity cannot be neglected, which can be seen from the theoretical predictions and reported experimental results achieved by using highly accurate methods [41]. It can be seen from Figure 6c that both the theoretical and experimental results show the water TO coefficient nonlinearity. Because of the small value of the TO coefficient nonlinearity and the limitation caused by the dynamic range or the background noise, the nonlinearity has not been reported in the experimental results of current SPR thermometers [12,13,22,23,24], and is hardly observed by other reported optical temperature sensors [5,6,7,8,9,10]. To demonstrate the feasibility of the PWR thermometry for accurate TO coefficient sensing in a wide temperature range, the experimental resonance angle is fitted linearly to the theoretical RI as shown in Figure 6d. It can be seen that the sensor setup responds to the temperature dependent RI variation linearly as it does in the RI test. This indicates that the PWR thermometer can measure the TO coefficient accurately so it can keep the measurement accuracy for the temperature sensing in the wide temperature range.

### 3.3. Real Time Natural Cooling Process Measurement

**Figure 7:**
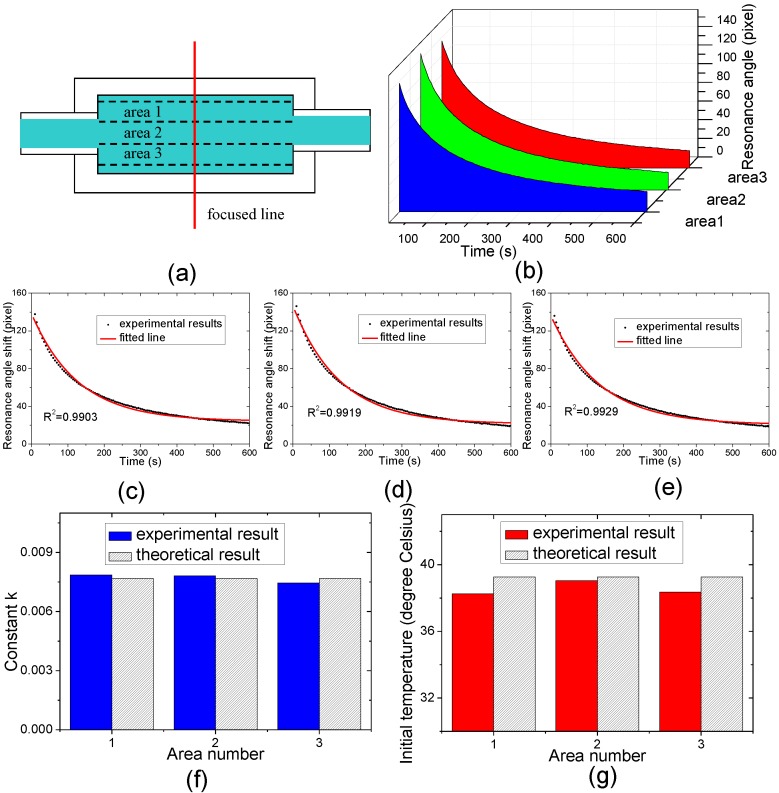
(**a**) Areas of interest marked with areas 1, 2, 3 are at the top, middle and bottom along the focused line in the cell respectively; (**b**) Waterfall plots of water cooling curves in the areas 1, 2, 3; (**c**–**e**) Experimental and fitted cooling curves of water cooling processes located in areas 1, 2, 3; (**f**) Comparison between the theoretical and experimental constants *k*; (**g**) Comparison between experimental and theoretical initial temperatures.

To demonstrate the feasibility of this system for real time temperature sensing, the natural cooling process of water is measured. The water at a temperature of 40 °C circulates through the flow cell continuously. Then we turn off the pump and the temperature control device in the water path. Considering the system response delay, we start to capture images at 10 s and stop at 600 s. The capture time interval is 5s. The final temperature is 30.1 °C measured by the thermocouple probe, and the room temperature is set to be 30 °C using the air conditioner. In this situation, the cooling process should follow Newton’s cooling law:

(13) $$\frac{dT}{dt}=-k(T-C)$$

(14) $$T=C+\left(T_{0}-C\right)e^{-kt}$$

where *T* is the water temperature at the time *t*, *T_0_* is the initial temperature, *k* is a constant, and *C* is the room temperature. The cooling process in the area of a hundred rows measured in the above experiments, which is located in the middle area of the flow cell and marked by “area 2” in Figure 7a, is analyzed as shown in Figure 7d. For reference analysis, the areas of the same hundred rows located on the sides of this area, which are marked with “area 1” and “area 3” in Figure 7a, are also analyzed and shown in Figure 7 c and e respectively. The cooling curves of water in three areas are shown by 3D waterfall plots in Figure 7b. The comparisons between the theoretical and experimental constant *k* and initial temperature *T_0_* are shown in Figure 7f,g, respectively.

It can be seen from Figure 7c–e that the experimental results are all well fitted to Newton’s cooling law. It also can be seen from Figure 7f that the constant *k* in the three areas is similar and close to the theoretical results. This demonstrates that the cooling processes can be measured by this thermometer in real time. The deviations may be caused by the change of thermal conduction mode and timing error in the measurement and the coefficient effect for different sensing channels.

It can be seen from the Figure 7g that the initial water temperatures in area 2 are slightly lower than the theoretical initial temperature of 39.26 °C calculated by Equation (14), which should be caused by the timing error. It can also be seen that the initial temperature at side areas, namely areas 1 and 3 are lower. Apart from the timing error, this deviation also could be caused by three factors. Firstly, due to the water flowing into and out of the flow cell through the holes in the middle of the side walls in this system, the water temperature in the middle area is easier to be controlled to the given temperature. Secondly, side areas are closer to the environment so they have a better heat exchange, whereas the middle area is surrounded by water with a large specific heat capacity, so the water temperature is hardly influenced by the environment temperature variations. Thirdly, there may be residual air in the flow cell, which stays at the side areas more easily due to the buoyancy and viscous force, which also has a negative effect on the temperature control in the side areas. Despite these facts, this system shows the ability to monitor temperature variations in real time. Apart from this, the cooling processes in different areas are measured simultaneously in this experiment, which indicates that this thermometer has the potential to be used for thermal gradient analysis.

## 4. Conclusions

We report on PWR (plasmon waveguide resonance) thermometry in this paper. The sPWR (symmetrical PWR) sensor, a kind of PWR sensors, is employed to demonstrate the feasibility experimentally. Owing to the enhancement of the evanescent field and the improvement of the extinction ratio in the reflectance spectrum, the sPWR sensor can achieve a RI resolution of 7.9 × 10^−7^ RIU. In the temperature sensing experiment, owing to the thermal shield effect of the dielectric layer and the improvement of the RI resolution, the PWR thermometer can achieve a temperature sensitivity of 9.4 × 10^−3^ °C, which is comparable to the recently reported high resolution optical temperature sensor [7]. Additionally, the TO coefficient nonlinearity is clearly observed using this thermometer, and it is so small that it is hardly measured by current optical thermometers. This indicates that the PWR thermometer has a wide dynamic range, and it may improve the measurement accuracy of optical thermometers in noninvasive temperature sensing applications. Finally, the cooling process monitoring shows that this method has the ability to monitor temperature in real time and the potential to be applied for thermal gradient analysis. Using the convention optical components and commercial coating techniques, the PWR thermometer could be easily implemented in practical applications, as the system has a simple optical arrangement and a good compatibility with a variety of SPR platforms, which have been used in diverse fields. PWR thermometry may promote the applications of optical temperature sensors and simulate further studies of new methods for noninvasive and real-time thermometry using electromagnetic fields.
